# Dual Radionuclide Therapy: The Synergistic Effects of [^161^Tb]Tb-PSMA and [^177^Lu]Lu-PSMA in Advanced Prostate Cancer Post [^177^Lu]Lu-PSMA Failure

**DOI:** 10.1007/s13139-024-00871-4

**Published:** 2024-07-10

**Authors:** Ahmed Saad Abdlkadir, Alaa Abufara, Akram Al-Ibraheem

**Affiliations:** 1 Department of Nuclear Medicine and PET/CT, King Hussein Cancer Center (KHCC), Al-Jubeiha, Amman 11941 Jordan; 2 Department of Medicine, King Hussein Cancer Center (KHCC), Al-Jubeiha, Amman 11941 Jordan; 3 School of Medicine, University of Jordan, Al-Jubeiha, Amman 11942 Jordan

**Keywords:** 177Lu-PSMA, 161Tb-PSMA, Dual radionuclide therapy, PSMA RLT, Metastatic castration resistant prostate cancer, mCRPC

Up to 30% of patients with metastatic castration-resistant prostate cancer (mCRPC) that is positive for prostate-specific membrane antigen (PSMA) on positron imaging do not respond to or develop resistance to monotherapy using [^177^Lu]Lu-labeled PSMA-targeted radioligands. For these challenging cases, dual radionuclide therapy combining [^225^Ac]Ac and [^177^Lu]Lu has been implemented, demonstrating both safety and efficacy. This interesting image details the initial experience with a tandem protocol of [^161^Tb]Tb/[^177^Lu]Lu-PSMA in an mCRPC patient following the failure of [^177^Lu]Lu-PSMA monotherapy.

Herein, we present a 74-year-old male with a 5-year history of stage IV prostate adenocarcinoma (Gleason score 4 + 4) underwent extensive treatment for more than 35 months, including radical prostatectomy, along with goserelin acetate, abiraterone, and enzalutamide therapies. The patient was classified as having mCRPC in the previous year, and he presented with elevated prostate-specific antigen (PSA) and alkaline phosphatase (ALP) levels of 321 ng/ml and 216 U/L, respectively. Baseline [^68^Ga]Ga-PSMA imaging confirmed eligibility for PSMA radioligand therapy (RLT), revealing widespread skeletal metastases (Fig. 1a-f, arrows) and a left seminal vesicle lesion (Fig. 1c, arrowhead). The patient received two cycles of [^177^Lu]Lu-PSMA RLT, dosed at 7.4 GBq for the first cycle (Fig. 1g) and similar for the second cycle (Fig. 1h), which did not yield clinical, molecular imaging (Fig. 1i), or biochemical improvements. Consequently, a multidisciplinary team recommended an alternative PSMA RLT strategy, administering 5.5 GBq of [^161^Tb]Tb-PSMA (Fig. 1j), followed by 7.4 GBq of [^177^Lu]Lu-PSMA two months later (Fig. 1k). This approach aimed to increase the linear energy transfer of [^161^Tb]Tb-PSMA via Auger electron emission in addition to enhancing the beta emission effect of the subsequent [^177^Lu]Lu-PSMA cycle. The treatment was well tolerated, had no side effects, and resulted in a partial imaging response (Fig. 1l-q) and significant reductions in the PSA and ALP levels (Fig. 1s), indicating a synergistic effect of the dual RLTs. Notably, PSA levels marked a downtrend from initial values of 321 ng/ml to 27 ng/ml at the end of therapy. Similarly, ALP experienced normalization, trending down from 216 IU/L to 75 IU/L (Fig. 1s).

**Fig. 1:**
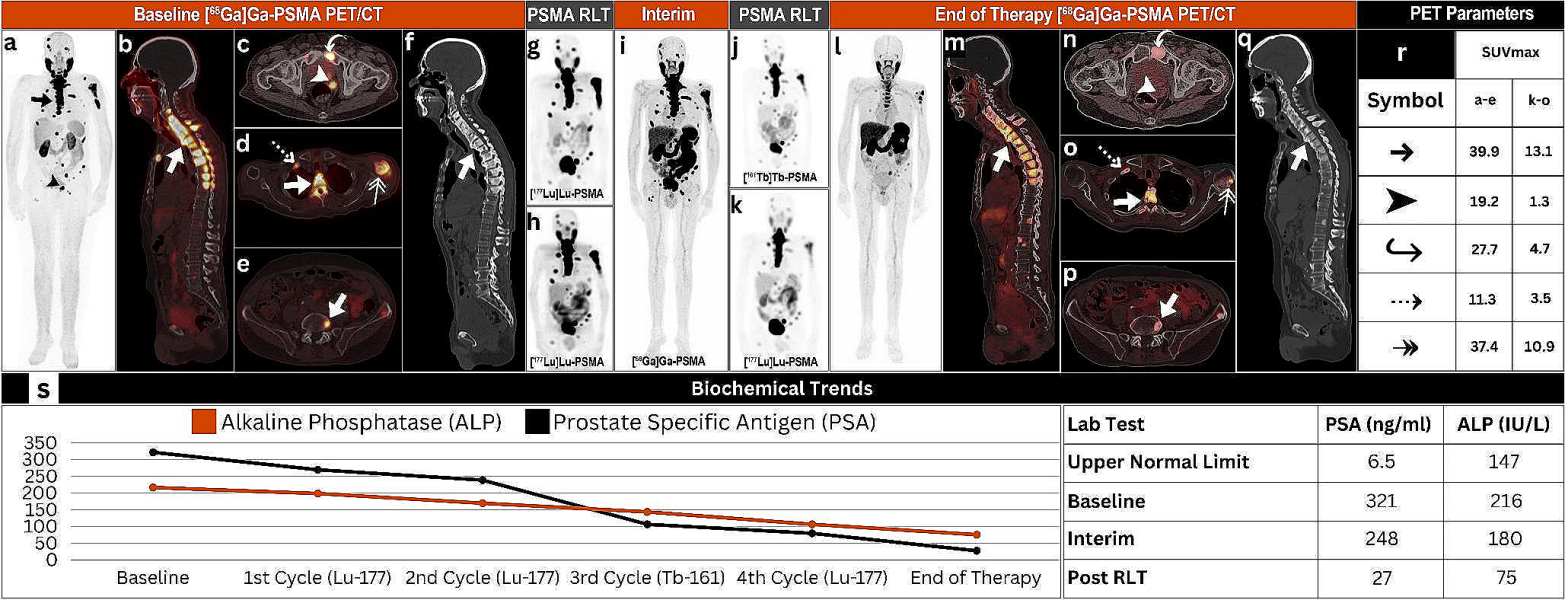
(**a**-**f**) Baseline [^68^Ga]Ga-prostate-specific membrane antigen (PSMA) positron emission tomography/computed tomography (PET/CT) demonstrates intense PSMA localization at sites of primary prostatic lesions and skeletal metastases (arrows). (**g**, **h**) Two cycles of [^177^Lu]Lu-PSMA, dosed at 7.4 GBq each, were administered with adequate scintigraphic localization. (**i**) Interim [^68^Ga]Ga-PSMA shows persistent viable disease. (**j**) A single dose of 5.5 GBq of [^161^Tb]Tb-PSMA followed by (**k**) 7.4 GBq of [^177^Lu]Lu-PSMA were administered with adequate scintigraphic localization. (**l**-**q**) Post-therapeutic [^68^Ga]Ga-PSMA highlighted partial disease control (arrows) (**r**) with favorable molecular imaging derived maximum standardized uptake values and (**s**) biochemical metrics achieved

As of now, only limited numbers of theranostic centers have access to [^161^Tb]Tb-PSMA [1]. While it shares similarities with [^177^Lu]Lu-PSMA, preliminary research suggests that [^161^Tb]Tb-PSMA may offer superior results due to its higher abundance of Auger and conversion electrons [2, 3]. Previous studies have demonstrated the safety and efficacy of using [^161^Tb]Tb-PSMA RLT as a standalone treatment in mCRPC patients [2, 4]. The combination of [^161^Tb]Tb/[^177^Lu]Lu-PSMA therapies has been considered for this case due to its potential for synergistic effect, offering a dual radionuclide therapy. Clinical trials are ongoing to explore its safety and efficacy [4, 5]. This case represents the first clinical experience where [^161^Tb]Tb-PSMA and [^177^Lu]Lu-PSMA were administered in a dual cycle regimen, resulting in short-term improvements in clinical outcomes and biochemical markers.

## Data Availability

The current study data are available from the corresponding author on reasonable request.

## Declarations

### Preprint Sharing

Not applied.
