# Correction to: Dual Radionuclide Therapy: The Synergistic Effects of [^161^Tb]Tb-PSMA and [^177^Lu]Lu-PSMA in Advanced Prostate Cancer Post [^177^Lu]Lu-PSMA Failure

**DOI:** 10.1007/s13139-024-00887-w

**Published:** 2024-10-29

**Authors:** Ahmed Saad Abdlkadir, Alaa Abufara, Akram Al-Ibraheem

**Affiliations:** 1 Department of Nuclear Medicine and PET/CT, King Hussein Cancer Center (KHCC), 11941 Al-Jubeiha, Amman, Jordan; 2 Department of Medicine, King Hussein Cancer Center (KHCC), 11941 Al-Jubeiha, Amman, Jordan; 3 School of Medicine, University of Jordan, 11942 Al-Jubeiha, Amman, Jordan

**Correction to Nuclear Medicine and Molecular Imaging (2024) 58:381–382**

10.1007/s13139-024-00871-4

The article Dual Radionuclide Therapy: The Synergistic Effects of [^161^Tb]Tb-PSMA and [^177^Lu]Lu-PSMA in Advanced Prostate Cancer Post [^177^Lu]Lu-PSMA Failure, written by Ahmed Saad Abdlkadir, Alaa Abufara, and Akram Al-Ibraheem, was originally published Online First without Open Access. After publication in volume 58, issue 6, pages 381–382, the author decided to opt for Open Choice and to make the article an Open Access publication. Therefore, the copyright of the article has been changed to © The Authors 2024 and the article is forthwith distributed under the terms of the Creative Commons Attribution (CC BY) license 4.0 International License, which permits use, sharing, adaptation, distribution and reproduction in any medium or format, as long as you give appropriate credit to the original author(s) and the source, provide a link to the Creative Commons licence, and indicate if changes were made. The images or other third party material in this article are included in the article’s Creative Commons licence, unless indicated otherwise in a credit line to the material. If material is not included in the article’s Creative Commons licence and your intended use is not permitted by statutory regulation or exceeds the permitted use, you will need to obtain permission directly from the copyright holder. To view a copy of this licence, visit http://creativecommons.org/licenses/by/4.0.

The original article has been corrected.

